# Sitagliptin, sitagliptin and metformin, or sitagliptin and amitriptyline attenuate streptozotocin-nicotinamide induced diabetic neuropathy in rats

**DOI:** 10.7555/JBR.26.20110054

**Published:** 2012-05-09

**Authors:** Ashish Kumar Sharma, Akash Sharma, Rita Kumari, Kunal Kishore, Divya Sharma, Bharthu Parthsarthi Srinivasan, Ashok Sharma, Santosh Kumar Singh, Samir Gaur, Vijay Singh Jatav, Prashant Sharma, Varnika Srivastava, Sneha Joshi, Megha Joshi, Prashant Kumar Dhakad, Davender Singh Kanawat, Akanksha Mishra, Anil Sharma, Dharmendra Singh, Ravinder Pal Singh, Himmat Singh Chawda, Rambir Singh, Sachin Kumar Raikwar, Muneem Kumar Kurmi, Pankaj Khatri, Ashutosh Agarwal, Arshee Munajjam

**Affiliations:** a Department of Pharmacology, Gyan Vihar School of Pharmacy, Suresh Gyan Vihar University, Jagatpura, Jaipur (Rajasthan) 302025, India;; b Department of Pharmacology, Delhi Institute of Pharmaceutical Sciences & Research, Pushpvihar, New Delhi 110017, India;; c Department of Pharmacology, Kota Collage of Pharmacy, RIICO Industrial Area, Ranpur, Kota (Rajasthan), Pincode 302025, India.

**Keywords:** diabetic neuropathy, nicotinamide-streptozotocin, metformin, pioglitazone, glimipiride, sitagliptin, amitriptyline

## Abstract

Diabetic neuropathies are a family of nerve disorders caused by diabetes. Symptoms of the disease include nerve palsy, mononeuropathy, mononeuropathy multiplex, diabetic amyotrophy, painful polyneuropathy, autonomic neuropathy, and thoracoabdominal neuropathy. In this study, type 2 diabetes in rats was induced with nicotinamide-streptozotocin. Drug treatment was initiated on the d 15, with the combination regimen of metformin, pioglitazone and glimipiride or metformin and sitagliptin or sitagliptin, amitriptyline and sitagliptin and led to significantly improved glycemic control, increased grip strength and paw jumping response on d 21, 28 and 35 (*P* < 0.001). Significant increases in blood protein levels and decreases in urinary protein levels were observed in the animals treated with the different regimens on d 21, 28 and 35 (*P* < 0.001). Combined treatment of streptozotocin and nicotinamide caused marked degeneration of nerve cells, while administration of metformin and sitagliptin showed tissue regeneration and no body weight gain. In conclusion, treatment with sitagliptin and sitagliptin combined with metformin or amitriptyline results in no body weight gain, but causes an increase in grip strength and pain sensitivity, exhibits neural protection, and reverses the alteration of biochemical parameters in rats with streptozotocin-nicotinamide induced type 2 diabetes.

## INTRODUCTION

Diabetic neuropathy occurs as a result of damages to the nervous system due to persistent hyperglycemia and can affect many parts of the body including the legs, feet, bladder, heart, gastrointestinal system, and reproductive system. The symptoms of diabetic neuropathy include pain, numbness, tingling, or prickling that begins in the feet. In the late stages of diabetic neuropathy, hands can be affected as well. In some cases of diabetic neuropathy, the abnormal sensations can extend to the arms, legs, and trunk. Relatively common conditions, which may be associated with diabetic neuropathy, include third nerve palsy, mononeuropathy, mononeuropathy multiplex, diabetic amyotrophy, polyneuropathy, autonomic neuropathy, and thoracoabdominal neuropathy. These conditions are thought to result from diabetic microvascular injury involving small blood vessels that supply nerves (*vasa nervorum*) in addition to macrovascular conditions that can culminate in diabetic neuropathy[1]-[3].

Physicians usually treat painful diabetic neuropathy with oral medications, although other types of treatments may help some people. People with severe nerve pain may benefit from a combination of medications or treatments[3]. Medications used to help relieve diabetic nerve pain include: 1) tricyclic antidepressants, such as amitriptyline, imipramine, and desipramine; 2) other types of antidepressants, such as duloxetine, venlafaxine, bupropion, paroxetine and citalopram; 3) anticonvulsants, such as pregabalin, gabapentin, carbamazepine and lamotrigine; 4) opioids and opioid-like drugs such as controlled-release oxycodone and tramadol, an opioid that also acts as an antidepressant[3].

Duloxetine and pregabalin are approved by the US Food and Drug Administration (FDA) specifically for treating painful diabetic peripheral neuropathy. Treatments that are applied to the skin, typically to the feet, include capsaicin cream and lidocaine patches. Studies suggest that nitrate sprays or patches for the feet may relieve pain. Studies of alpha-lipoic acid, an antioxidant, and evening primrose oil have shown that they can help relieve symptoms and may improve nerve function[3].

Dipeptidyl peptidase 4 (DDP-4) inhibitors, or glyptins, are a class of oral hypoglycemics that block DPP-4. The first agent of this class, sitagliptin, was approved by the FDA in 2006. Sitagliptin entered the Australian drug market in late 2007 for the treatment of difficult-to-control diabetes mellitus type 2. Another DPP-4 inhibitor, vildagliptin, was added to the Pharmaceutical Benefits Scheme (PBS) Program listings in 2010 on the basis of a similar cost-minimization basis to sitagliptin[4],[5]. Their mechanisms of action are thought to result from increased incretin levels [glucagon-like peptide-1 (GLP-1) and glucose-dependent insulinotropic polypeptide (GIP)], which inhibit glucagon release that increases insulin secretion, decreases gastric emptying, and decreases blood glucose levels[4]-[6].

Drugs belonging to this class are: 1) sitagliptin; 2) vildagliptin; 3) saxagliptin; 4) linagliptin, which is being developed by Boehringer Ingelheim; 5) dutogliptin, a phase III drug that is being developed by Phenomix; 6) gemigliptin, which is being developed by LG Life Sciences; 7) alogliptin, which is developed by Takeda[4]-[6]. Berberine, a common herbal dietary supplement, also inhibits DDP-4, which at least partly explains its anti-hyperglycemic activities[6].

DPP-4 inhibitors are enzyme inhibitors that inhibit the enzyme DPP-4 and are a potent treatment for type 2 diabetes. Inhibition of the DPP-4 enzyme prolongs and enhances the activity of incretins that play an important role in insulin secretion and blood glucose control regulation. Type 2 diabetes is a chronic metabolic disease that can be caused by pancreas β-cell dysfunction, deficiency in insulin secretion, insulin resistance and/or increased hepatic glucose production. It is one of the fastest growing health concerns in the world. Current treatments are often inefficient at sustaining glycemic control and may cause undesirable side effects, such as weight gain and episodes of hypoglycemia. Therefore, new and more effective drugs have been developed with DPP-4 inhibitors playing a significant role[4]-[6].

Sitagliptin has a novel structure with β-amino amide derivatives. Sitagliptin has shown excellent selectivity and *in-vivo* efficacy, which encouraged researchers to inspect the new structure of DPP-4 inhibitors with appended β-amino acid moiety. Further studies are being developed to optimize these compounds for the treatment of diabetes. Crystallographic structure of sitagliptin along with molecular modeling has been used to continue the search for structurally diverse inhibitors. A new potent, selective and orally bioavailable DPP-4 inhibitor was discovered by replacing the central cyclohexylamine in sitagliptin with 3-aminopiperidine. A 2-pyridyl substitution was the initial structure-activity relationship (SAR) breakthrough since the group plays a significant role in potency and selectivity for DPP-4. Sitagliptin works to competitively inhibit DPP-4. This enzyme breaks down the incretins GLP-1 and GIP, gastrointestinal hormones that are released in response to a meal. By preventing GLP-1 and GIP inactivation, they are able to increase the secretion of insulin and suppress the release of glucagon. This drives blood glucose levels towards normal. As the blood glucose level approaches normal, the amounts of insulin released and glucagon suppressed diminish. This tends to prevent the “overshoot” and subsequent hypoglycemia, which is seen with some other oral hypoglycemic agents[4]-[6]. The present study was designed to assess the effects of sitagliptin, and sitagliptin combined with metformin or amitriptyline on streptozotocin-nicotinamide induced diabetic neuropathy in rats.

## MATERIALS AND METHODS

### Animals and drugs

Adult Wistar Albino rats, weighing 150 to 250 g, which were bred in the Animal House, Gyan Vihar School of Pharmacy, Suresh Gyan Vihar University, were used. All animal experiments were approved by the Institutional Animal Ethical Committee (IAEC-III, Gyan Vihar School of Pharmacy), and Committee for the Purpose of Control and Supervision of Experiments on Animals (CPCSEA, No. 1234/a/08/CPCSEA). The animals were housed in polycarbonate cages in a room with a 12/12 h day/night cycle, at a temperature of 24±2°C and at a humidity of 45% to 64%. During the study period, animals were fed with a balanced commercial diet and water *ad libitum*.

Streptozotocin was purchased from Alexis Biochemicals (San Diego, CA, USA); Nicotinamide was supplied by Ottokemi (Mumbai, India); sitagliptin phosphate (100 mg) was purchased from Merch Sharp and Dohme (Pavia, Italy), amitriptyline hydrochloride (25 mg) from Wockharot Ltd. (Solan, India), metformin hydrochloride (500 mg) from Wallace Pharmaceutical. (Solan, India), pioglitazone hydrochloride (15 mg) from Emcure Pharmaceutical (Pune, India), glimepiride (2 mg) from C. B. Healthcare (Maharashtra, India), and citric acid from Central Drug House (New Delhi, India), respectively. Streptozotocin was dissolved in citrate buffer (0.1 mol/L, pH 4.5) and nicotinamide was dissolved in normal saline before use.

### Induction of diabetic neuropathy

Non-insulin-dependent diabetes mellitus was induced in overnight-fasted rats by a single intraperitoneal injection of 65 mg/kg streptozotocin, 15 min after the intraperitoneal administration of 110 mg/kg nicotinamide. Hyperglycemia was confirmed by the elevated glucose levels in plasma, determined at 72 h and then on d 7 after injection. The animals with blood glucose concentration of more than 200 mg/dL were used for the study[7],[8].

### Treatment protocol

Type 2 diabetes was induced by intraperitoneal administration of streptozotocin and nicotinamide. Insulin resistant rats were randomly assigned to different groups with 6 animals per group. Group I: the normal control group; Group II: the negative control group (streptozotocin-nicotinamide induced type 2 diabetes); Group III: the standard treatment group in which the animals received 120 mg/kg metformin in combination with 1.25 mg/kg pioglitazone and 0.7 mg/kg glimepiride[9]; Group IV: in which the animals received the combination treatment of 120 mg/kg metformin and 11.67 mg/kg sitagliptin; Group V: in which the animals received the combination treatment of 120 mg/kg amitriptyline and 11.67 mg/kg sitagliptin; Group VI: in which the animals received 11.67 mg/kg sitagliptin only. Drug treatment was given daily for 35 d *via* oral catheter in the morning. On d 7, 14, 21, 28 and 35, blood glucose level, body weight, muscular grip strength, hot plate-induced thermal pain, blood protein level and urinary protein level were evaluated in rats with streptozotocin-nicotinamide induced diabetic neuropathy.

### Effects of drug treatment on body weight, grip strength and pain sensitivity in rats

The body weights of all diabetic animals were measured weekly during the study period[10]. Grip strength during diabetes was evaluated using the Rotarod apparatus. The test was used to assess muscle strength or neuromuscular function in rodents, which could be influenced by sedative drugs, muscle relaxant compounds and toxic agents. The apparatus consists of a 3 cm diameter horizontal wooden or metal rod coated with rubber with and attached to a motor with the speed adjusted to 25 r/min. The rod is 23 cm in length and is divided into three sections of discs, thereby allowing simultaneous testing of three rats. Cages below the section serve to restrict the movement of the animals from the roller. Only those animals which demonstrated the ability to remain on the revolving rod for at least 1 min were used for the test. Every week the rats were placed on the rotating rod. The time to fall was measured using a single dose of sitagliptin and its combinations with other drugs[11].

The sensory function was assessed by evaluating pain threshold. The hot plate test was carried out according to the method of Eddy's *et al*[12]. Animals were placed on hot plates maintained at 55±1°C, and the reaction time was recorded as response latency. The response latencies were measured before and after treatment. The cut-off time for hot-plate latency was 10 s.

### Determination of biochemical indicators

Blood glucose level was measured weekly using a glucometer following the administration of the testing compounds. The biuret end-point assay (Span Diagnosis Ltd., Surat, India) was used to detect total blood protein[13]. Rat urine was collected and the protein was precipitated by trichloroacetic acid to a final concentration of 0.33 mol/L. After the mixture was kept at room temperature for 30 min, the precipitants were centrifuged at 110 *g* for 20 min. The precipitant was processed and, after reaction with biuret reagent, absorbency was measured using a colorimeter[14]. The total protein concentrations in blood and urine were determined using the following formula: Total protein concentration (g/dL) = Absorbance of test/Absorbance of standard ×6.5.

### Histopathological examination of the sciatic nerve

Histopathological examination of the sciatic nerve was done before and after treatment. Wistar Albino rats were used as a mammalian model for histopathological observation of the sciatic nerve. In preparation for surgery, rats were anesthetized by intraperitoneal ketamine (80 mg/kg)[9], and maintained under sedation with additional boluses of ketamine for the duration of each individual experiment. Once anesthetized, animals were placed in the prone position and the right and left sciatic nerves were exposed over the length of the femur. An incision was made posterior-laterally extending from the gluteus muscles to the popliteal region. This allowed access to the sciatic nerve from its pelvic cavity exit to the level of the knee and visualization of specific motor branches (number of fibularis and number of tibialis) to the biceps femoris, gastrocnemius, and distal muscles. This surgical procedure served to expose sufficient area for electrical and optical stimulation and electrical recording of compound muscle action potentials (CNAPs) along the nerve and compound muscle action potentials (CMAPs) from the biceps femoris and gastrocnemius muscles. The muscle fascia overlying the nerve was carefully removed to expose the nerve surface with its epineurial covering maintained intact. Removal of this fascia greatly decreases energy required for stimulation. Nerves were continually moistened with normal saline to avoid desiccation during the acute study. The typical stimulation of rat sciatic nerve in this study was approximately 2 mm in diameter. The typical fascicle thickness was constant across all mammalian species (although the number of fascicles per nerve varied greatly) and tended to be between 200 and 400 µm[15].

### Statistical analysis

All values were expressed as mean±SEM, and all statistical analyses were performed using Sigma Stat 3.5 and Sigma Plot 10.0 (Sigma). Differences of variables before and after treatment were tested for statistical significance using paired Student's *t* test, and the differences between different groups were compared using one-way analysis of variance (ANOVA) followed by Tukey-Kramer multiple comparison test. *P* values < 0.05 were considered statistically significant.

## RESULTS

### Sitagliptin in combination with metformin or amitriptyline has no effect on the body weight of diabetic rats

The body weight decreased rapidly in rats with streptozotocin-nicotinamide induced diabetic neuropathy. Measurement of the body weight of rats in all experimental groups is shown in ***Table 1***. Significant decreases in body weight were observed in streptozotocin-induced diabetic rats on week 1 post injection (147.5±4.958 g in non-diabetic rats *vs* 131.67±3.005 g in diabetic control rats at 1 week after streptozotocin treatment, *P* < 0.01). A progressive loss of body weight was noted from week 0 to week 5 in the diabetic control rats (147.5±4.958 g at week 0 *vs* 90.17±2.836 at week 5, *P* < 0.001). The body weight of the animals in the other groups also decreased significantly until d 21, when compared with the negative control group. On d 15, animals treated with standard protocol (500 mg metformin in combination with 15 mg pioglitazone and 2 mg glimipiride), metformin in combination with sitagliptin (100 mg), sitagliptin in combination with amitriptyline (25 mg), and sitagliptin did not induce any apparent body weight gain.

**Table 1:** Effects of sitagliptin and its combinations on body weight (gm) of rats.

Group	Day-1	Day-7	Day-14	Day-21	Day-28	Day-35
Control	131.67 ± 6.412	140.83 ± 6.350	149.17 ± 6.380	164.17 ± 5.833	180.00 ± 4.655	195.00 ± 4.830
Neg. Control	147.50 ± 4.958	131.67 ± 5.780**	110.83 ± 3.005**	102.50 ± 3.096**	98.50 ± 2.140**	90.17 ± 2.836**^ab^
Stan. Group	146.70 ± 5.110	127.50 ± 8.441**	108.33 ± 5.725**	104.17 ± 4.729**	104.17 ± 4.729**	104.17 ± 4.729**^##^
Met. +Sita.	158.33 ± 9.369	141.67 ±10.382**	123.33 ± 10.462**	120.83 ± 10.362**^a^*	120.83 ± 10.362**^##^	120.83 ± 10.362**^##^
Ami. +Sita.	138.33 ± 8.408	122.50 ± 8.441**	102.50 ± 6.158**	97.50 ± 5.284^b##^	97.50 ± 5.280*^a^**	97.50 ± 5.284^b##^
Sita.	148.33 ± 6.146	130.83 ±5.689**	109.17 ± 4.729**	103.33 ± 3.073^b##^	103.33 ± 3.073**^##^	103.33 ± 3.073**^##^

Compared to the normal control group, **P < 0.01; b = not significant (ns) when compared to the normal control group; Compared to the negative control group, ^##^*P* < 0.01; c = ns when compared to the standard group. Neg: negative; Stan: standard treatment with metformin+pioglitazone+glimipiri de; Met: metformin; Ami.: amitriptyline; Sita: sitagliptin.

(mean±SEM, *n* = 6)

### Sitagliptin in combination with metformin or amitriptyline significantly reduces blood glucose level in diabetic rats

The blood glucose levels of rats in all experimental groups, except the normal control group, increased significantly after the streptozotocin-nicotinamide injection until d 14 (***Table 2***). On d 21, 28 and 35 post induction, significantly increased blood glucose levels were detected in the negative control group, compared with the normal control group (*P* < 0.001). In the diabetic control group (negative control), blood glucose increased to the peak level of 315±4.472 mg/dL on d 35 and was found to be significantly increased (*P* < 0.001) compared with the value on d 1 (85.33±2.376 mg/dL). Control animals remained normoglycemic during the entire testing period of 35 d. Treatment with standard protocol (combination of 500 mg metformin and 15 mg pioglitazone and 2 mg glimipiride), metformin in combination with sitagliptin (100 mg), sitagliptin in combination with amitriptyline (25 mg), and sitagliptin alone on d 15 exhibited significantly decreased blood glucose levels compared with those in the normal control group on d 21, 28 and 35 (*P* < 0.001) (***Table 2***).

**Table 2:** Effects of sitagliptin and its combinations on blood glucose level (mg/dL) in rats.

Group	Day-1	Day-7	Day-14	Day-21	Day-28	Day-35
Control	86.00 ± 1.713	86.00 ± 1.317	86.83 ± 1.600	86.50 ± 0.992	85.67 ± 0.871	86.00 ±1.033
Neg. Control	85.33 ± 2.376	178.15 ±7.000**	271.00 ± 3.120**	283.17 ±2.810**	295.83 ± 2.301	315.00 ± 4.470**
Stan. Group	87.67 ± 1.783	182.00 ±4.502**	288.00 ± 3.468**	232.83 ±3.859**	192.50 ± 3.939^a^**	141.83 ± 6.030**^##^
Met. +Sita.	87.67 ± 1.453	185.33 ±1.926**	284.17 ± 5.706**	225.83 ±3.736**	178.83 ± 3.229**^##^	173.33 ± 3.116**^##^
Ami. +Sita.	88.17 ± 1.327	194.17 ±4.729**	282.17 ± 4.285**	236.50 ±3.344**	190.67 ± 1.085**^##^	176.83 ± 1.721^b##^
Sita.	86.17 ± 1.740	188.50 ±5.536**	281.50 ± 3.519**	242.50 ±4.703**	188.33 ± 2.140**^##^	174.67 ± 3.169**^##^

Compared to the normal control group, **P < 0.01; b = not significant (ns) when compared to the normal control group; Compared to the negative control group, ^##^*P* < 0.01; c = ns when compared to the standard group. Neg: negative; Stan: standard treatment with metformin+pioglitazone+glimipiri de; Met: metformin; Ami.: amitriptyline; Sita: sitagliptin.

(mean±SEM, *n* = 6)

### Sitagliptin in combination with metformin or amitriptyline improves muscular grip strength of diabetic rats

Measurement of muscular grip strength was used to evaluate diabetic neuropathy after 14 d of streptozotocin-nicotinamide injection. Muscular grip strength was reduced significantly in all rats with streptozotocin-induced diabetes. In the normal control group, muscular grip strength was normal (60±4.683 *vs* 63±3.581 N), and no statistically significant difference was found (*P* > 0.05), while significant difference of muscular grip strength was found in the diabetic control group (59.33±3.029 *vs* 8.00±0.894 N, *P* < 0.001). The grip strength of all treated animals increased significantly compared with the positive control group on d 28 and 35 (*P* < 0.001). The grip strength of rats treated with standard protocol and combination of amitriptyline and sitagliptin was significantly greater on d 28 and 35, when compared with the negative control group (***Table 3***).

**Table 3:** Effects of sitagliptin and its combinations on muscle grip strength (s) in rats.

Group	Day-1	Day-7	Day-14	Day-21	Day-28	Day-35
Control	60.00 ± 4.683	63.50 ± 2.683	61.38 ±2.671	59.00 ±3.176	65.00 ± 4.638	63.00 ± 3.581
Neg. Control	59.33 ± 3.029	34.00 ± 1.862**	23.50 ± 1.310**	17.67 ± 1.116**	12.50 ± 1.147**	8.00 ± 0.894**
Stan. Group	58.17 ± 4.262	33.00 ± 2.683**	22.67 ± 0.843**	28.83 ± 0.872**	39.83 ± 0.830**^##^	47.00 ± 0.954**^b##^
Met. +Sita.	58.50 ± 2.907	34.50 ± 1.893**	25.50 ± 1.335**	28.33 ± 0.980**	37.78 ± 1.493**^##c^	42.67 ± 1.085**^##c^
Ami. +Sita.	57.17 ± 3.673	33.17 ± 2.167**	26.00 ± 1.461**	30.67 ± 1.145**	34.33 ± 1.493**^##^	51.37 ± 1.085^b##c^
Sita.	57.33 ± 3.670	33.50 ± 1.668**	25.00 ± 1.438**	28.38 ± 1.282**	32.28 ± 1.282**^##c^	40.00 ± 1.2.2^b##c^

Compared to the normal control group, **P < 0.01; b = not significant (ns) when compared to the normal control group; Compared to the negative control group, ^##^*P* < 0.01; c = ns when compared to the standard group. Neg: negative; Stan: standard treatment with metformin+pioglitazone+glimipiri de; Met: metformin; Ami.: amitriptyline; Sita: sitagliptin.

(mean±SEM, *n* = 6)

### Effects of sitagliptin and in combination with metformin or amitriptyline on pain sensation (thermal pain)

In rats, a single systemic injection of streptozotocin-nicotinamide induced a hyperalgesic reaction observed on d 14 after the onset of diabetic neuropathy. In this study, hyperalgesic reaction was evaluated for a period of 14 d post treatment with streptozotocin and nicotinamide. The paw jumping response was measured by the Eddy's hot plate. There was significant difference in paw jumping response 14 d post induction of diabetic neuropathy in rats, but there was no significant difference found in the control group in which diabetes was not induced (5.33±0.51 *vs* 5.83±0.75). In diabetic rats (negative control), there was significant increase in paw jumping response (5.5±0.54 *vs* 13.67±1.38). The paw jumping responses of all treated rats on d 21, 28 and 35 were reduced significantly compared with the negative control group (***Table 4***). On d 28, the paw jumping response of rats treated with metformin in combination with pioglitazone and glimipiride, metformin in combination with sitagliptin, and amitriptyline in combination with sitagliptin was found to be significantly different from that in the normal control group. Combined treatment of amitriptyline and sitagliptin on d 15-35 caused significant effect in pain threshold compared the negative control group.

**Table 4:** Effects of sitagliptin and its combinations on thermal pain (s) in rats.

Group	Day-1	Day-7	Day-14	Day-21	Day-28	Day-35
Control	5.33 ± 0.51	5.50 ± 0.54	5.45 ± 0.54	5.66 ± 0.51	5.83 ± 0.75	5.68 ± 0.78
Neg. Control	5.50 ± 0.54	8.50 ± 0.61	9.65 ± 1.03**	10.50 ± 0.83**	12.66 ±1.03**	13.67 ± 1.38**
Stan. Group	5.33 ± 0.51	8.38 ± 0.83	9.45 ± 1.03**	8.16 ± 0.75^##b^	7.00 ± 0.98^b##^	6.23 ± 1.38^##^
Met. +Sita.	5.65 ± 0.65	8.66 ± 0.51*	10.23 ± 0.81**	8.20 ± 1.22**^##c^	7.33 ± 0.51**^##c^	6.34 ± 0.78**^b##^
Ami. +Sita.	5.50 ± 0.54	8.33 ± 0.51	9.66 ± 0.81**	10.16 ± 0.75**^##c^	11.23 ± 0.63^b##c^	12.38 ± 0.91**^b##c^
Sita.	5.50 ± 0.54	8.50 ± 0.54	9.83 ± 0.75**	7.83 ± 0.75^##bc^	7.23 ± 0.81^b##c^	6.45 ± 1.08^b##^

Compared to the normal control group, **P < 0.01; b = not significant (ns) when compared to the normal control group; Compared to the negative control group, ^##^*P* < 0.01; c = ns when compared to the standard group. Neg: negative; Stan: standard treatment with metformin+pioglitazone+glimipiri de; Met: metformin; Ami.: amitriptyline; Sita: sitagliptin.

(mean±SEM, *n* = 6)

### Effects of sitagliptin in combination with metformin or amitriptyline on blood and urinary protein levels

The blood protein levels in all experimental groups, except the normal control group, were significantly decreased while the urinary protein excretion was increased after streptozotocin-nicotinamide injection (***Table 5***). On d 21, 28 and 35 post induction, statistically significant decrease in blood protein level and increase in urinary protein level in the negative control group were observed in comparison with the normal control group (*P* < 0.001) (***Table 5*** and ***Table 6***). In the diabetic group (negative control), the blood protein level decreased from the maximum value of 6.28±0.034 *vs* 3.30±0.081 mg/dL and the urinary protein level increased from 0.24±0.035 *vs* 3.41±0.023 mg/dL, and statistically significant differences were observed (*P* < 0.001). However, normal blood protein level was observed in the control group (6.27±0.044 *vs* 6.25±0.037 mg/dL) during the period of the study (***Table 5***). Significantly increased blood protein level and decreased urinary protein level were detected in the animals treated with different treatment protocols, compared with the negative control group on d 21, 28 and 35 (*P* < 0.001). In addition, the blood protein level was 4.82±0.006 mg/dL in rats treated with metformin-sitagliptin combinations on d 35, which was significantly different from that in the negative control group.

**Table 5:** Effects of sitagliptin and its combinations on blood protein level (mg/dL) in rats.

Group	Day-1	Day-7	Day-14	Day-21	Day-28	Day-35
Control	6.27 ± 0.044	6.27 ± 0.048	6.25 ± 0.040	6.25 ± 0.042	6.25 ± 0.010	6.25 ± 0.037
Neg. Control	6.28 ± 0.035	4.76 ± 0.042**	4.15 ± 0.016**	3.89 ± 0.009**	3.59 ± 0.019**	3.30 ± 0.018**
Stan. Group	6.28 ± 0.046	4.75 ± 0.024**	4.14 ± 0.012**	4.33 ± 0.008**	4.57 ± 0.034**^##^	4.79 ± 0.009**^##^
Met. +Sita.	6.30 ± 0.024	4.75 ± 0.019**	4.16 ± 0.018**	4.35 ± 0.005**	4.55 ± 0.009**	4.82 ± 0.016**^##^
Ami. +Sita.	6.30 ± 0.027	4.79 ± 0.027**	4.17 ± 0.020**	4.28 ± 0.027**	4.51 ± 0.046**^##^	4.70 ± 0.020^b##^
Sita.	6.29 ± 0.012	4.81 ± 0.018**	4.17 ± 0.009**	4.33 ± 0.015**	4.48 ± 0.040**^##c^	4.78 ± 0.020^##^

Compared to the normal control group, **P < 0.01; b = not significant (ns) when compared to the normal control group; Compared to the negative control group, ^##^*P* < 0.01; c = ns when compared to the standard group. Neg: negative; Stan: standard treatment with metformin+pioglitazone+glimipiride; Met: metformin; Ami.: amitriptyline; Sita: sitagliptin.

(mean±SEM, *n* = 6)

**Table 6:** Effects of sitagliptin and its combinations on protein level in urine (mg/dL) in rats.

Group	Day-1	Day-7	Day-14	Day-21	Day-28	Day-35
Control	0.27 ± 0.014	0.25 ± 0.018	0.30 ± 0.021	0.26 ± 0.052	0.25 ± 0.010	0.25 ± 0.042
Neg. Control	0.24 ± 0.035	1.76 ± 0.032**	2.15 ± 0.026**	2.89 ± 0.009**	3.09 ± 0.017**	3.47 ± 0.023**
Stan. Group	0.23 ± 0.046	1.79 ± 0.025**	2.24 ± 0.012**	1.43 ± 0.008**	1.23 ± 0.034**^##^	1.09 ± 0.007**^##^
Met. +Sita.	0.30 ± 0.024	1.85 ± 0.019**	2.26 ± 0.028**	1.35 ± 0.008**	1.05 ± 0.009**^##^	0.98 ± 0.026**^##^
Ami. +Sita.	0.30 ± 0.027	1.91 ± 0.047**	2.27 ± 0.070**	1.26 ± 0.017**	1.01 ± 0.046**^##^	0.93 ± 0.060^b##^
Sita.	0.29 ± 0.012	1.83 ± 0.017**	2.21 ± 0.017**	1.36 ± 0.017**	1.20 ± 0.080**^##c^	1.03 ± 0.060**^##^

Compared to the normal control group, **P < 0.01; b = not significant (ns) when compared to the normal control group; Compared to the negative control group, ^##^*P* < 0.01; c = ns when compared to the standard group. Neg: negative; Stan: standard treatment with metformin+pioglitazone+glimipiri de; Met: metformin; Ami.: amitriptyline; Sita: sitagliptin.

(mean±SEM, *n* = 6)

### Histopathology of sciatic nerve

**Fig. 1:**
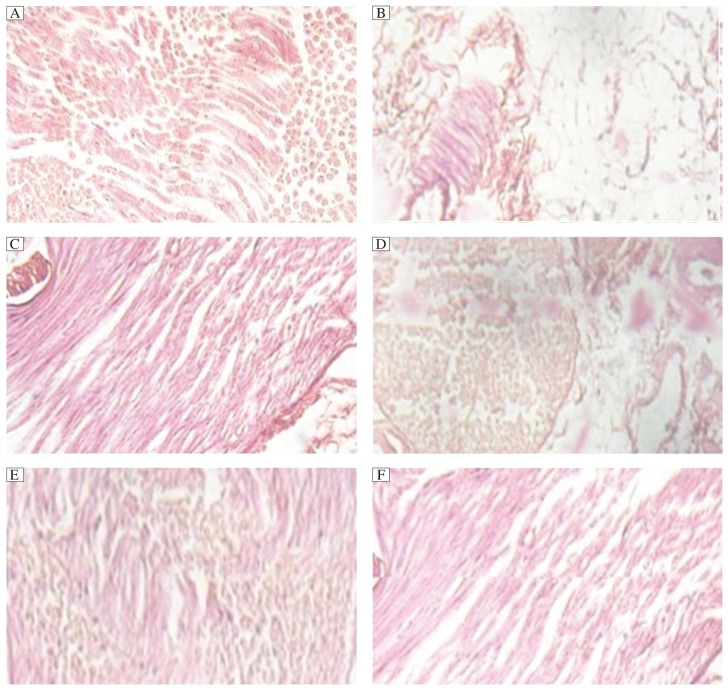
Histopathological examination of the sciatic nerve in different groups. A: Cross-sectional profile of the sciatic nerve showed normal structure in rats treated with normal saline. B: significant degeneration of nerve cells in rats treated with a combination of streptozotocin and nicotinamide. C: normal growth in rats treated with a combination of metformin, pioglitazone and glimipiride. normal growth in rats treated with metformin combined with sitagliptin. Normal growth in rats treated with amitriptyline-sitagliptin combination (E) and significant growth in rats treated with sitagliptin (F) were observed.

Histopathological examination of the sciatic nerve revealed that the nerve cells of the streptozotocin-nicotinamide treated group showed marked degenerations. Increases in tissue regeneration capacity were observed in rats treated with standard protocol, while normal cell growth in tissues was found in the normal control group. In addition, combined treatment of metformin and sitagliptin showed significant tissue regeneration capacity when compared with the control group as well as the standard treatment group (***Fig. 1***).

## DISCUSSION

The present study investigated the effects of sitagliptin and sitagliptin combined with other drugs on streptozotocin-nicotinamide induced diabetic neuropathy in rats. Streptozotocin is most commonly used to induce diabetes in experimental animals and administration of streptozotocin-nicotinamide caused diabetic neuropathy[16],[17], probably due to destruction of the β-cells of the islets of Langerhans of the pancreas, overproduction of blood glucose and decreased utilization by tissues, which form the fundamental basis of hyperglycemia in diabetes mellitus. Hyperglycemia accompanied by weight loss is seen in adult rats treated with streptozotocin-nicotinamide and is stable for weeks, indicating irreversible destruction of β-cells of the islets of Lengerhans of the pancreas. Sitagliptin has been shown, in several clinical studies to improve metabolic control in type 2 diabetes, both as monotherapy or in combination with metformin, sulfonylurea, thiazolidinediones or insulin. The reduction in HbA1c is approximately 0.6% to 1.0% from the baseline levels of 7.5% to 8.7% over 6 to 12 months of therapy. Sitagliptin has a favorable safety profile and is highly tolerable, and there is a minimal risk of hypoglycemia. Furthermore, sitagliptin is body weight neutral or induces a slight body weight reduction. Sitagliptin may be used in the early stages of type 2 diabetes in combination with metformin or other treatments in subjects with inadequate glycemic control on these treatments alone. Sitagliptin may also be used in monotherapy or in combination with insulin in more advanced stages of the disease[16],[17].

Sitagliptin is a DPP-4 inhibitor, which is believed to exert its actions in patients with type 2 diabetes by slowing the inactivation of incretin hormones. Concentrations of the active intact hormones are increased by sitagliptin, thereby increasing and prolonging the action of these hormones. Incretin hormones, including GLP-1 and GIP, are released by the intestine throughout the day, and their levels are increased in response to a meal. These hormones are rapidly inactivated by the enzyme, DPP-4. The incretins are part of an endogenous system involved in the physiologic regulation of glucose homeostasis. When blood glucose concentrations are normal or elevated, GLP-1 and GIP increase insulin synthesis and release from pancreatic β-cells by intracellular signaling pathways involving cyclic adenosine monophosphate. GLP-1 also lowers glucagon secretion from pancreatic α-cells, leading to reduced hepatic glucose production. By increasing and prolonging active incretin levels, sitagliptin increases insulin release and decreases glucagon levels in the circulation in a glucose-dependent manner. Sitagliptin demonstrates selectivity for DPP-4 and does not inhibit DPP-8 or DPP-9 activity *in vitro* at concentrations approximating those from therapeutic doses[16],[17]. GLP-1 is an enteric hormone secreted in the small intestine in response to nutrient ingestion. Interest in GLP-1 is based on its ability to amplify glucose-stimulated insulin secretion. In contrast to agents that promote insulin secretion via glucose-independent mechanisms, the dependence of GLP-1 on glucose concentration is considered a unique safety advantage due to a lower risk of hypoglycemia. However, the utility of GLP-1 is limited by its short half-life (2 min) secondary to rapid degradation by the proteolytic enzyme DPP-4, which exerts its biological effects via two mechanisms. First, DPP-4 enzymatic activity is exhibited through the membrane-spanning form of the molecule and the circulating soluble form. DPP-4 preferentially cleaves substrates with an amino-terminal proline or alanine at position, including GLP-1. Second, DPP-4, independent of its enzymatic properties, binds to adenosine deaminase and conveys intracellular signals *via* dimerization and activation of intracellular pathways. The prevention of GLP-1 degradation through the inhibition of the DPP-4 enzyme is emerging as a therapeutic strategy to enhance GLP-1 activity. The administration of sitagliptin enhances the ability of GLP-1 to produce insulin in response to elevated concentrations of blood glucose, to inhibit the release of glucagon following meals, to slow the rate of nutrient absorption into the bloodstream, to slow the rate of gastric emptying, and to reduce food intake[16],[17].

GLP-1 and GIP play an important physiological role in regulation of blood glucose levels[6],[9]. Meal ingestion stimulates the release of incretins, which stimulate insulin synthesis and release (GLP-1 and GIP), suppress glucagon release (GLP-1), delay gastric emptying (GLP-1), and increase satiety (GLP-1)[16]-[18]. The effects to enhance insulin and suppress glucagon levels are glucose-dependent, and only observed when glucose levels are normal or elevated, and not seen when glucose levels are low. These mechanisms attenuate the post-meal rise in glucose, and contribute to lowering fasting glucose concentrations. These glucoregulatory effects of GLP-1 and GIP are short-lived due to rapid inactivation by DPP-4[18]-[20]. DPP-4 inhibitors enhance incretin action by blocking their degradation and, hence, inactivation. Inhibition of DPP-4 extends the half-life and increases the concentrations of circulating intact (active) GLP-1 and GIP. In patients with type 2 diabetes, DPP-4 inhibition leads to higher levels of active incretins, both during fasting and post meal, which in turn lead to higher insulin release, lower glucagon levels, and improved fasting and post meal glucose concentrations. Patients with type 2 diabetes have reduced post meal GLP-1 concentrations with normal GLP-1 action. Thus, DPP-4 inhibition addresses one defect that may contribute to hyperglycemia in this disease. Following meal ingestion, intact (active) GLP-1 and GIP are released from gut endocrine cells and function to lower blood glucose levels by stimulating glucose-dependent insulin release from pancreatic β-cells (GLP-1 and GIP) and suppressing glucose-dependent glucagon release from pancreatic α-cells (GLP-1). Several orally active DPP-4 inhibitors have now been developed to treat type 2 diabetes, including sitagliptin, vildagliptin and saxagliptin, and sitagliptin was approved in the United States in October, 2006 for the treatment of patients with type 2 diabetes.

Many patients with type 2 diabetes remain inadequately treated, in part because of limitations of existing anti-hyperglycemic therapies that are associated with adverse effects such as hypoglycemia or weight gain. DPP-4 inhibitors represent a valuable new addition to the therapeutic armamentarium to treat patients with type 2 diabetes, providing practitioners with a well-tolerated management option for improving 24-h glycemic control by engaging glucose-dependent physiological processes. Whether chronic treatment will be associated with improvements in β-cell function that can alter the natural history of diabetes remains an important, unanswered question—one that must be addressed in long-term clinical studies. Sitagliptin protects renal ischemia reperfusion induced renal damage in diabetes[25]. Amitriptyline relieves diabetic neuropathy pain in patients with normal or depressed mood. In a randomized, double-blind crossover study, 29 patients with painful diabetic neuropathy received 6 weeks of amitriptyline and 6 weeks of an “active” placebo that mimicked amitriptyline side effects. Amitriptyline was superior to placebo in relieving pain in weeks 3 through 6. Both steady, burning pain and lancinating pains were relieved[26]. Sitagliptin 100 mg once daily added to ongoing metformin therapy is efficacious and well tolerated in patients with type 2 diabetes that has inadequate glycemic control with metformin alone[27]. DDP-4 inhibitors improve glycemic control in patients with type 2 diabetes mellitus when used as monotherapy or in combination with other anti-diabetic drugs (metformin, sulphonylurea, or thiazolidinedione). Saxagliptin added to metformin therapy is effective in improving glycemic control in patients with type 2 diabetes mellitus inadequately controlled by metformin alone. In addition, saxagliptin plus metformin is non-inferior to sitagliptin plus metformin, and is generally well tolerated[28].

Sitagliptin prevents the development of metabolic and hormonal disturbances, increased β-cell apoptosis and liver steatosis induced by a fructose-rich diet in normal rats. Maiztegui and colleagues[29] tested the effect of sitagliptin and exendin-4 upon metabolic alterations, β-cell mass decrease and hepatic steatosis induced by fructose in rats. The administration of exendin-4 and sitagliptin to animals prevented the development of all the metabolic disturbances and the changes in β-cell mass and fatty liver. Thus, these compounds, which were useful for treating type 2 diabetes, would prevent/delay the progression of early metabolic and tissue markers of this disease. DDP-4 inhibitors improve glucose homeostasis in type 2 diabetics by inhibiting degradation of the incretin hormones. DDP-4 inhibition also prevents the breakdown of the vasoconstrictive neuropeptide Y and, when angiotensin-converting enzyme (ACE) is inhibited, substance P. Marney *et al*.[30] provided the first evidence for an interactive hemodynamic effect of DDP-4 and ACE inhibition in humans.

Diabetic neuropathy is a long-term complication of diabetes that develops early in the course of the disease and is observed in 60%-70% of all diabetic patients. It is known that diabetic neuropathy is a nerve degenerative disease characterized by axonal degeneration, nerve fiber demylination, and a reduction in the number of medium-to-large diameter nerve fiber, particularly in peripheral nerve. Diabetic neuropathy is triggered by hyperglycemia, which leads to a persistent accelerated flux of glucose through the polyol pathway. The rate-limiting enzyme in this pathway is aldose reductase. The increased flux through the polyol pathway is followed by abnormal protein kinase C metabolism, oxidative stress, accelerated glycation, and decreased endoneural capillary perfusion, leading eventually to nerve degeneration. In this study, the hypoglycemic effect was observed with the treatment of sitagliptin and its combination with other drugs in streptozotocin-nicotinamide induced hyperglycemic rats, with the maximum effect seen post treatment with the sitagliptin and metformin combination, which may be due to its synergestic antidiabetic effect. Induction of diabetic neuropathy with streptozotocin-nicotinamide is also associated with characteristic loss of body weight, which is due to increased muscle wasting and loss of tissue protein. Diabetic rats treated with sitagliptin and combined with the other two drugs showed no body weight gain when compared to the diabetic control, which may be due to its effect in controlling muscle wasting. The presence and severity of diabetic neuropathy has been shown to be associated with decreased muscle strength in diabetes mellitus. The current study observed significant improvement in motor behavior, in particular grip strength in diabetic rats after treatment with sitagliptin and its combination with other drugs. Treatment with amitryptyline-sitagliptin combinations caused significant increase in grip strength compared with the diabetic control group, while significant decrease in grip strength was observed in the negative control group.

Hyperalgesia is a constant feature of sensory dysfunction in spontaneous and experimental models of diabetic neuropathy. Our findings indicate that the improvement in hot-plate response related to the increased pain threshold of diabetic animals treated with sitagliptin-amitryptyline combinations. The response in the sitagliptin and amitryptyline treated group was found to be better than in other treatments. The analgesic effect was similar to the normal control after treatment with metformin combined with pioglitazone and glimipiride and sitagliptin in combination with metformin. Significant increase in pain threshold was observed in the combined treatment of sitagliptin and amitryptyline. The result confirms the usefulness of amitryptyline combined with sitagliptin in symptomatic treatment of painful diabetic neuropathy.

Numerous studies have reported that, in diabetic neuropathy, blood protein levels decrease and excretion of protein from the urine increases. This is consistent with the findings in our study diabetic control group rats. Following treatment with metformin in combination with pioglitazone and glimipiride and sitagliptin, blood protein level in this group was approximately normal. The lower blood protein level observed in the negative control group may be due to its metabolism and excretion from the urine. Treatment with amitryptline in combination with sitagliptin or metformin and sitagliptin combination significantly reduced urinary protein excretion, compared with the negative control group. It has been reported that the peripheral nerve becomes ischemic and hypoxic due to osmotic shrinkage or retardation of normal axonal maturation in streptozocin-nicotinamide treated rats. Cross-sectional profiles of sciatic nerves of rats in the diabetic group showed significant degeneration of nerve fibers, which is consistent with previously reported findings. Combined treatment of sitagliptin and metformin showed normal sciatic nerve growth, compared with the normal control group.

In conclusion, the significant effect of sitagliptin and its combination with other drugs on diabetic neuropathy in rats was observed. This may be attributed to the synergistic and potentiating action of the combined treatment since they contain a diverse array of active principles, which are able to target multiple mechanisms involved in the pathophysiology of diabetic neuropathy. Treatment of sitagliptin only and in combination with metformin or amitriptyline causes no weight gain and an increase in grip strength and pain sensitivity, which indicates neural protection. Administration of sitagliptin alone and combined treatment of sitagliptin and metformin or amitriptyline reverses the alteration in biochemical parameters, causes morphological changes in sciatic nerve and improves the general behavioral parameters in diabetic rats induced by streptozocin-nicotinamide.
